# Liability Determination of School Sports Injury Accidents: An Analysis Framework Based on Evolutionary Game

**DOI:** 10.3390/ijerph16183403

**Published:** 2019-09-13

**Authors:** Qing Lan, Xiaojun Li

**Affiliations:** 1 School of Public Administration, Central South University, Changsha 410083, China; jxlanqing@csu.edu.cn; 2 College of Physical Education, Yichun University, Yichun 336000, China

**Keywords:** injury accidents, liability determination, evolutionary game model, strict liability rule, proportional liability rule

## Abstract

In recent years, the outbreak of many school sports injury accidents aroused widespread public concern about liability determination of accident. Previous studies have examined the legal application of the liability principles from a law perspective, but few kinds of research attempted to analyze the progress of liability determination from the perspective of “law economics”. To fill this research gap, we introduce the evolutionary game model, as an important theoretical tool of “law economics”, to investigate how various factors influence the strategy selection of the parties, as well as examine what liability principle can effectively treat school sports injury accidents. The results indicate that the strategic selection of the subject of liability is significantly related to the accident compensation cost and the prevention cost of both parties. Moreover, we also find that both strict and proportional liability rules can play key roles in dealing with the issue of liability determination of school sports injury accidents, but the two liability rules have different effects on the strategic selection of parties. More specifically, compared to the strict liability principle, the proportional liability principle can motivate both the school and the students to adopt the active strategy of “appropriate caution” to prevent occurring sports injury accidents in schools.

## 1. Introduction

The all-round development of students, including moral education, intellectual education, physical education, artistic education, and labor education, is the leading idea of school education in China [1]. Under the guidance of this educational thinking, physical education has aroused widespread attention from the public. In fact, in 2016, the General Office of the State Council has emphasized that school should strengthen physical education and extracurricular exercise, and ensure the time and effect of students’ sports activities [2]. Furthermore, the Ministry of Education announced that some indicators, such as the opening rate of physical education classes, the training of sports programs, as well as the regular organization of sports games, etc., would be considered in physical education supervision and evaluation system [3]. Despite those laws and policies have provided the system safeguards for the development of school physical education; however, in practice, sports injury accidents still occurred frequently in the process of school physical education. Taking Beijing as an example, 171 cases of civil litigation disputes caused by injuries in physical education teaching and extracurricular physical activity entered the field of judicial relief through litigation by the parties, requesting the court to determine the liability between 2000 and 2010 [4].

School sports injury accident mainly refers to the accident in which students suffer physical and mental damage caused by the physical activities inside and outside the school (including physical education, sports competitions, and after-school sports training), which is organized and implemented by the school, as well as the sports venues or sports facilities that the school is responsible for managing [5,6]. Experience shows that effective preventive measures to school sports injury accidents are not only related to the personal safety and healthy growth of students but also related to the development of physical education activities in schools. However, at present, sports injury accidents in schools show a significant trend for an increase, which also often caused a large number of compensation disputes (medical expenses, disability and compensation fees, etc.), and the new difficulty in determining accident liabilities are frequently sued to the court [7,8]. According to statistics, 68.6% of the plaintiffs in the 121 cases of sports injury accidents judged by courts at all levels in Beijing filed a claim for mental compensation [9]. Additionally, another investigation with 203 cases also indicated that the principle of fault liability was the legal basis for the majority of liability determination and the schools undertook 60% of the liability for injury accidents [10]. Furthermore, Wang (2016) showed that injury liability identification is an important way to settle the disputes of physical education in schools, maintain the right of physical education, property right, and personal right, regulate school physical education behavior, as well as curb the decline of school physical education quality and students’ physical fitness [11]. In this context, the school should not only provide relief for the students according to the law but also protect the legitimate rights and interests of the school and guarantee the order of physical education activities. Therefore, to balance the interests between schools and the students, it is helpful to explore the imputation of school sports injury disputes to promote the healthy development of school sports.

## 2. Literature Review

Since the 1980s, there has been abundant related research that studies the liability distribution of school physical education accidents. The existing literature explains the causes of school sports injury accidents mainly include human-related factors, material-related factors, social-related factors, management-related factors, and comprehensive factors [12]. Furthermore, Fang (2018) integrated social-related factors, management-related factors, as well as comprehensive management factors into the external factors [13]. Regarding the injury liability identification, Yan (2017) indicated that the liability of school sports injury accidents could be divided into five forms, namely, school liability, students liability, third-party liability, multi-party liability, and liability caused by force majeure. However, according to a recent survey with 58 judicial precedents of school sports injury accidents, the proportion of school fault and compensation was often higher than that of other responsible subjects [14]. In fact, China often treat the school sports injury accidents based on the general principles of the Civil Law of the People’s Republic of China to solve the problem of civil liability [15]; the Measures for the Safe Handling of Student Accidents is often applicable for the problem of administrative liability [16]; and the Criminal Law of the People’s Republic of China is used to handle criminal liability [17]. However, Li et al. (2018) indicated that the way to deal with school sports safety accidents in China is still not strong by the law, and the legislative foundation is not deep, which make it difficult to decide whether or not have laws to follow when a school injury accident occurs [18].

The key to the liability of sports injury accidents lies in which principle would be to use for the legal ruling. In fact, in China’s judicial practice, the principles of imputation for school sports injury accidents mainly include fault liability, fault presumption, fair liability, no-fault liability, and multiple liabilities, of which the principle of fault liability emphasized that the liability determination should be based on the mistake responsibility; namely, liability is assumed when there is a fault, and no-fault is no liability. The principle of fault presumption refers to that the injuring party can only exempt himself from tort liabilities when he can provide rebuttal evidence to prove that he is not negligent [19]. The principle of fair liability means that the parties are not at fault and by the principle of fairness and reasonableness, the parties share the liability. The principle of no-fault liability means that no matter whether the party is at fault or not, the party should bear the liability stipulated by the law. The principle of multiple liabilities refers to the determination of a general liability principle should be based on the applicable conditions of a tort, and other liability principles are supplemented to determine the liability for injury accidents. Han (2009) indicated that in school sports injury accidents, the principle of fair liability could be taken into account based on the principle of fault liability, and the principle of fault presumption and no-fault principle should be applied when there was a special statutory infringement [20]. In fact, most scholars also agree with the imputation principle of fault liability. For example, Yu (2002) took typical cases as research samples and shown that the liability determination of students’ injury accidents in physical education classes should be based on the principle of fault liability, supplemented by the principle of fair liability [21,22]. Li (2012) also took the similar views and indicated that the principle of fault liability should be acted as the main imputation principle of sports injury accidents of primary and secondary school students, but should judge whether the students have the civil capacity and make appropriate adjustments [23]. Furthermore, Tan et al. (2011) developed a fault liability-oriented liability system supplemented by the risk-taking principle when considering the particularity of school sports activities [24]. Additionally, from the point of law practice, Wang (2016) also indicated that the traditional principle of dualistic imputation, namely, the fault liability is the main part, and the loss is shared fairly, is disadvantageous to the comprehensive and in-depth development of sports activities in colleges and universities [25].

The above-mentioned literature mainly emphasized how to settle the dispute of imputation from the perspective of law, which can not only make it conform to the legal standard, but also realize the fair value goal of the law. In summary, the existing literature mainly explored theoretical issues such as the protection of participants’ rights and interests, the feasibility and legal basis of solving sports injury accidents disputes, and few studies attempt to investigate the measurement of interests in the judicial settlement of sports injury disputes [26]. In fact, the operation of laws and the allocation of resources often follow the principle of the minimum transaction cost, that is, the value of law operation lies in the minimization of social costs and the realization of the optimal allocation of resources [27]. Additionally, Fang (2018) also indicated that the occurrence of student injury accidents and the public thinking of the school to bear “unlimited liability” had become the important factors of restricting the implementation of school physical education policy [13]. Additionally, Liu (2018) in his research on the adjudicated cases of sports injury disputes, pointed out that the court mainly measured the interests of the parties and the institution, and there were mainly two kinds of “conflict theory” and “reconciliation theory.” However, at present, the scope of interests measured is still relatively narrow, the position of the court is not clear, and there is also no unified standard of interest measurement [26]. Therefore, some scholars have realized that the imputation process of school sports injury accident can be considered as a process of interest measurement, which is in accordance with the hypothetical of rational economic man. That is, people‘s codes of conduct are based on the pursuit of minimal effort to get the maximum return [28,29]. Therefore, the hypothesis of rational economic man in economics can be appropriately introduced to analyze the costs and benefits of the operation of laws and policies and try to maximize the overall interests of the society.

As a kind of tort liability, school sports injury accidents have a strong two-way attribution effect. That is to say, if we pay more attention to avoiding injury to students, it will increase the liability of the school and make the school suffer losses. On the contrary, if the focus is on reducing the damage to the school, it will infringe on the interests of the students. In this case, the essence of solving the infringement problem of school sports injury accident is to balance the interests of the school and students and their guardians, to avoid serious damage between them [30]. Therefore, we can model the relationship in the imputation progress of a school sports injury accident as a game process between the school concerned and the injured students and their guardians on the division of accident liability, of which both parties of the game are concerned about maximizing their interests, and the school, as the main responsible party of sports injury accidents, often hopes to bear as little liability as possible. That is, once the law determined that the school should bear the corresponding liability, which would promote the school to take more stringent measures to prevent the occurrence of sports injury accidents, such as increasing the investment of sports facilities and reducing sports activities and so on. However, for the injured students and their guardians, they often tend to claim more compensation, which means that they may take irrational measures, such as besieging schools, media exposure, and so on to safeguard their rights and interests, once the compensation is not satisfied. Additionally, it is also a continuous interactive and game process. Namely, when the public’s fierce claim behavior threatens the interests of the school, which make the school will increase the compensation and get a settlement with the parents, or adopt legal means to protect its rights and interests. Then the public will respond to the strategy change of the school accordingly. Thus, we can conclude that strategy selection, in essence, is often a dynamic process of continuous learning and adjustment in the decision-making process of school and parent co-creation, which means that evolutionary game analysis is more suitable for the game situation of both parties.

Motivated by the above discussion, this article tried to investigate the imputation progress of school sports injury accident from the perspective of an evolutionary game based on law economics, different from previous literature focusing on the perspective of law. In fact, previous studies have rarely involved in this aspect, especially ignoring the value consideration of the maximization of interests of the parties, which involves what principle of liability can achieve a win-win situation between the parties, and this is exactly the challenge to solve such disputes in judicial practice. In this context, this study provided a new reference to understand the liability determination of school sports injury accidents, as well as provided a new analytical framework to examine the behavioral strategies of the parties in school sports injury accidents. More specifically, we aimed to investigate the key factors affecting the decision-making of both parties of sports accidents in schools, analyzed the strategic choice of both parties in school sports injury accidents, and analyzed the efficiency of the principle of application of the law. To achieve this goal, first, we considered the factors that influence the strategic selection of the subject of liability in school sports accidents. Second, we established an evolutionary game model to simulate the evolutionary game process of accident imputation. Finally, we provided an analysis framework to simulate and compare the efficiency of various applicable principles of law in the game of school sports accidents imputation.

## 3. Methodology

Game theory, which derived from decision theory [31], was a good choice to explore situations characterized by strategic interactions among multiple stakeholders and has been widely applied in various fields [32,33]. With the deepening study in the field of campus safety accidents, game models as mathematical tools can also be used to quantitatively investigate the imputation process of campus safety accidents. In fact, the division of liabilities during campus safety accidents is a game process among stakeholders, such as the school, students, and their guardians, etc. In the short-term, each stakeholder would tend to ignore the maximization of the overall interests for pursuing the maximum of one’s interests. However, in the long-term, the achievement of overall interests during campus safety accidents largely depends on their collaboration. That is, both the school and student adopt the “appropriate caution” strategy during school campus accidents. Additionally, in the long-term, the school’s avoiding-liability strategy in campus safety accidents is not always a static state, but a constantly changing process. This means that their stable strategy cannot be achieved by one-off adjustment from stakeholders, but these game participants gradually adjust their strategy to obtain the equilibrium of the system. Therefore, in our article, the evolutionary game model can be used as a tool to investigate the imputation progress during campus safety accidents.

Evolutionary game theory originated from classical game theory and was mainly used to explore how each participant in the game can choose their interests through learning and imitation in the competitive activities, and how to formulate these strategies. The participants in the game can be an individual or an organization. That is to say, under the premise of limited rationality, each participant in the game cannot achieve game equilibrium through one-time strategic choice. On the contrary, through multiple continuous games, all participants use learning and imitation to find their optimal strategies to achieve the steady-state of the entire system.

### 3.1. Analysis Framework

For sports injury accidents in schools, the problem of liability is the evolutionary game process between the school and the injured student and their guardians, of which the school is the main responsible party of sports injury accidents and instinctively tends to eliminate the evidence against itself to reduce the amount of compensation and even achieve the purpose of non-compensation. Students and their guardians, as the potential victims of sports injury accidents, would tend to take irrational measures, such as besieging schools, media exposure, etc., to obtain more compensation once an accident occurs. However, in practice, the high frequency of sports injury accidents may cause the two parties to play a continuous game of accident liability, which means that the two participants of the game constantly adjust and improve the strategy according to changing factors, and influence the opponent’s action strategy to achieve a dynamic balance. Therefore, in the long run, the attributed liability of sports injury accident is essentially a pattern gradually formed by the two participants during the long-term interactive gaming process. For example, once the court determines that the school in charge of the accident bears all liability. High compensation costs often encourage the school to implement measures that may limit sports activities in the future, resulting in students being unable to carry out normal sports activities, which are not conducive to the improvement of the quality of physical education in schools. However, in this context, students can obtain individual compensation, which makes students prone to have a certain degree of security negligence.

To sum up, the internal mechanism of attributed liability of sports injury accidents and evolutionary game theory has a strong fit. It is of theoretical significance and practical value to use evolutionary game theory to explore the strategic choice law, dynamic evolution path, and influencing factors of each participant in the process of attributed liability.

### 3.2. Evolutionary Game Model

In light of the frequent occurrence of sports injury accidents in schools, the rationality of accident liability is conducive to promoting the healthy development of school physical education. According to the evolutionary game theory, both parties of the school injury accident are bounded rational players, which means that they select their strategy based on the comparison of their cost-benefit, but because it is impossible to find the optimal strategy immediately, the individuals within the group choose to emulate the advanced member behavior in the group and eventually achieve equilibrium state.

#### 3.2.1. The Bounded Rational Players in the Game

There are two interested players in the game model of the liability principle for sports injury accidents in schools. One is the school in an accident, and another is students and their guardians.

As the main responsible party of sports injury accidents, the school must relieve students according to the law, and it also protects the legitimate rights and interests of the school and guarantee the order of physical education activities. Additionally, the students and their guardians, as the primary victim of the accident, must not only seek compensation for accidents, but also protect their legitimate rights and interests, and pursue the right to participate in sports activities.

#### 3.2.2. The Impact Factor

The essence of attributed liability of sports injury accidents in schools is that under the legal framework, the two parties of the accident take the maximization of their interests as the starting point and make rational choices based on relevant impact factors.

(1) Prevention cost. Given the appropriate investment in sports safety prevention, it can greatly reduce the occurrence of sports injury accidents and avoid sports injury accident disputes, so it is important to explore the cost of prevention. In practice, the cost of prevention of sports injury accidents in schools mainly includes the cost of safety education, the cost of examination and maintenance of sports facilities, the cost of training sports teachers, and the cost of monitoring students’ physical fitness.

(2) Compensation cost. The allocation of liability for sports injury accident is the main impact factor for the two participants to adjust the game strategy. In the case of sports injury accidents in schools, the cost of compensation mainly includes the cost of accident losses (such as the personal and property losses of injured students) and the cost of accident implementation (such as litigation costs in the process of compensation disputes, court operating expenses, and time and energy).

#### 3.2.3. Game Strategy

In many dispute cases of school sports injury accidents, the court determines the liability of school sports injury accidents mainly rely on the principle of fault liability, due to lack of targeted, precise and effective legal provisions That is, compensation for fault, no-fault no compensation [34]. However, in most practices, the treatment of imputation that takes into account the fair value of the law would increase the school’s liability in sports injury accidents. If the school is not satisfied with the distribution of legal liability, in the long run, it will tend to take more prudent measures to prevent accidents, such as reducing school sports activities, reducing the difficulty of sports activities, and prohibiting sports activities. However, those prudent measures would inhabit students to enjoy the fun of sports, which further restricts the development of physical education in schools. In this article, we hypothesize that the two participants will choose to treat school sports activities with an appropriate cautious or not cautious attitude. That is, the strategy set of both parties can be set as follows: school (appropriate caution, no caution), injured students and their guardians (appropriate caution, no caution). It should be noted that appropriate caution refers to general or common-sense tasks of attention.

#### 3.2.4. Function Set

We assume that the probability of the school choosing the “appropriate caution” strategy is α, the probability of choosing a “no caution” strategy is 1−α; the probability that students and their guardians choose an “appropriate caution” strategy is β, and the probability of selecting a “no caution” strategy is 1−β. $C_{c}$ refers to the cost of preventing sports injury accidents when the school adopts the “appropriate caution” strategy (including student safety education fees, inspection and maintenance costs for sports facilities, physical education teacher training fees, student physical fitness monitoring fees). $C_{s}$ refers to the cost of prevention when the students take the “appropriate caution” strategy ($c_{s}<c_{c}$), and when adopting the “no caution” strategy, the prevention costs of schools and students are minimal, we set it to zero. Additionally, we assume that the probability of a sports injury accident is minimal (for simplicity, we set it as zeros) when both participants adopt “appropriate caution”. On the contrary, as long as any participant does not keep caution, there will be a school sports injury accident. Thus, the school would undertake the loss cost of the accident (such as the personal injury and property loss of the injured student) and the execution cost of the accident (such as litigation costs in the process of compensation disputes, court operating costs, and time cost). Furthermore, we assume the school should bear the cost of an accident is ap, where a is the proportion of compensation for accident liability (0≤a≤1) and p is the total loss of the accident. b refers to the proportion of the prevention costs of students and their guardians undertaken by the school when students and their guardians adopt (no caution, appropriate caution) strategy (0≤b≤1. Thus, we construct a game matrix between the two participants, as shown in Table 1.

## 4. Equilibrium Analysis

### 4.1. The Replicated Dynamic Equations

$E_{a}$ and $E_{n}$ refer to the expected gains of the school of choosing “appropriate caution” and “no caution” strategies, respectively; $S_{a}$ and $S_{n}$ refer to the expected gains of the students and their guardians of choosing “appropriate caution” and “no caution” strategies, respectively, and $E_{c}$ and $S_{c}$ refer to the average expected gains of the school and the students and their guardians respectively. According to game theory, we calculated the gain matrix of the players as follows:

The expected gains of the school of choosing the “appropriate caution” strategy are:

(1) $$\begin{array}{ll} E_{a} & =-\beta c_{c}-(1-\beta )c_{c}\\ & =-c_{c} \end{array}$$

Additionally, the expected gains when the school chooses the “no caution” strategy are:

(2) $$E_{n}=-\beta \left(p+bc_{s}\right)-\left(1-\beta \right)ap$$

According to Equations (1) and (2), we can obtain the replicated dynamic equation when the school chooses the “appropriate caution” strategy:

(3) $$\begin{array}{ll} F\left(\alpha \right) & =\frac{d\alpha }{dt}=\alpha \left(E_{a}-E_{c}\right)\\ & =\alpha \left(1-\alpha \right)\left(E_{a}-E_{n}\right)\\ & =\alpha \left(1-\alpha \right)\left(\beta \left(\left(1-a\right)p+bc_{s}\right)-\left(c_{c}-ap\right)\right) \end{array}$$

The expected gains of the students and their guardians of choosing the “appropriate caution” strategy are:

(4) $$S_{a}=-\alpha c_{s}-\left(1-\alpha \right)\left(1-b\right)c_{s}$$

Additionally, the expected gains of the students and their guardians of choosing the “no caution” strategy are:

(5) $$S_{n}=-\alpha p-\left(1-\alpha \right)\left(1-a\right)p$$

Similarly, according to Equations (4) and (5), we can also obtain the replicated dynamic equation when the students and their guardians choose the “appropriate caution” strategy:

(6) $$\begin{array}{ll} G\left(\beta \right) & =\frac{d\beta }{dt}=\beta \left(S_{a}-S_{c}\right)\\ & =\beta \left(1-\beta \right)\left(S_{a}-S_{n}\right)\\ & =\beta \left(1-\beta \right)\left[\left(p\left(1-a\right)-\left(1-b\right)c_{s}\right)-\left(bc_{s}-ap\right)\alpha \right)] \end{array}$$

To sum up, we can get the equilibrium solution of the accountability game system by analyzing the replicated dynamic Equations (3) and (6):

(7) $$\{\begin{array}{c} \alpha^{*}=\frac{p\left(1-a\right)-\left(1-b\right)c_{s}}{bc_{s}-ap}\\ \beta^{*}=\frac{c_{c}-ap}{\left(1-a\right)p+bc_{s}} \end{array}$$

Based on the results from Equation (7), we can conclude that $\left(\alpha^{*},\beta^{*}\right)$ is the equilibrium point to judge the evolution path of attributed liability game model. That is to say, under certain conditions, the change direction of the equilibrium point affects the strategy choice of both participants in the accident, and then affects the final stability of the system. First, as far as the school in the accident concerned, the probability of choosing an “appropriate caution” strategy is significantly related to accident losses, the proportion of liability determinations, and student prevention costs, of which the higher the accident loss, the school will more likely adopt an “appropriate caution” strategy. On the contrary, if the cost of prevention is high, the school will tend to choose “no caution” strategy as its optimal strategy. Second, in terms of the injured students and their guardians, the probability of choosing the “appropriate caution” strategy is significantly related to the prevention cost of both parties, the loss of the accident and the proportion of liability, of which the higher the cost of safety prevention, the injured students and their guardians will more likely adopt an “appropriate caution” strategy. On the contrary, when the cost of prevention is higher, the injured students and their guardians will tend to adopt the “no caution” strategy as their optimal strategy.

### 4.2. Stability Analysis of Evolutionary Game System

By solving above-mentioned replicated dynamic equations, we can obtain five equalization points $p\left(0,0\right),p\left(0,1\right),p\left(1,0\right),p\left(1,1\right),p\left(\alpha^{*},\beta^{*}\right)$ in the two-dimensional space {(α,β)|0≤α,β≤1}. Since the equilibrium solution is not necessarily a partial equilibrium solution, this section will take the Jacobian matrix and its stability analysis method to estimate the stability of the above points. The Jacobian matrix is as follows:

(8) $$J=\left[\begin{array}{cc} \left(1-2\alpha \right)\left[\beta \left(\left(1-a\right)p+bc_{s}\right)+\left(ap-c_{c}\right)\right] & \alpha \left(1-\alpha \right)\left(\left(1-a\right)p+bc_{s}\right)\\ \beta \left(1-\beta \right)\left(\left(1-a\right)p-\left(1-b\right)c_{s}\right) & \left(1-2\beta \right)\left[\left(\left(1-a\right)p-\left(1-b\right)c_{s}\right)-\left(bc_{s}-ap\right)\alpha \right] \end{array}\right]$$

Then we can obtain the matrix determinant and matrix trace, respectively:

(9) $$Det\left(J\right)=\frac{\partial F\left(\alpha \right)}{\partial \alpha }\frac{\partial G\left(\beta \right)}{\partial \beta }-\frac{\partial F\left(\alpha \right)}{\partial \beta }\frac{\partial G\left(\beta \right)}{\partial \alpha }$$

(10) $$Tr\left(J\right)=\frac{\partial F\left(\alpha \right)}{\partial \alpha }+\frac{\partial G\left(\beta \right)}{\partial \beta }$$

According to the above formulas, we can calculate the determinant and trace of four equilibrium points of O(0,0), A(0,1), B(1,1), and C(1,0). Table 2 shows the results:

Let $\pi_{1}=ap-c_{c},\pi_{2}=p+bc_{s}-c_{c},\pi_{3}=p-c_{s},\pi_{4}=\left(1-a\right)p-\left(1-b\right)c_{s}$. $\pi_{1}$ is the net income of the school of adopting the “appropriate caution” strategy when the students and their guardians adopt the “no caution” strategy. $\pi_{2}$ is the net income of the school of adopting the “appropriate caution” strategy when the students and their guardians adopt the “appropriate caution” strategy. $\pi_{3}$ is the net income of the students and their guardians of adopting an “appropriate caution” strategy when the school adopts the “appropriate caution” strategy. $\pi_{4}$ is the net income of the students and their guardians of adopting the “appropriate caution” strategy when the school adopts the “no caution” strategy. Referring to the decision method of the evolutionary stable strategy (ESS) proposed by Friedman in 1991 [35], when the system satisfies the condition as Det(J)>0 and Tr(J)<0 at a certain equilibrium point, this equilibrium point is the stable evolution strategy of the system. Then, we can obtain the stability discrimination table, including ten scenarios, as shown in Table 3.

**Figure 1:**
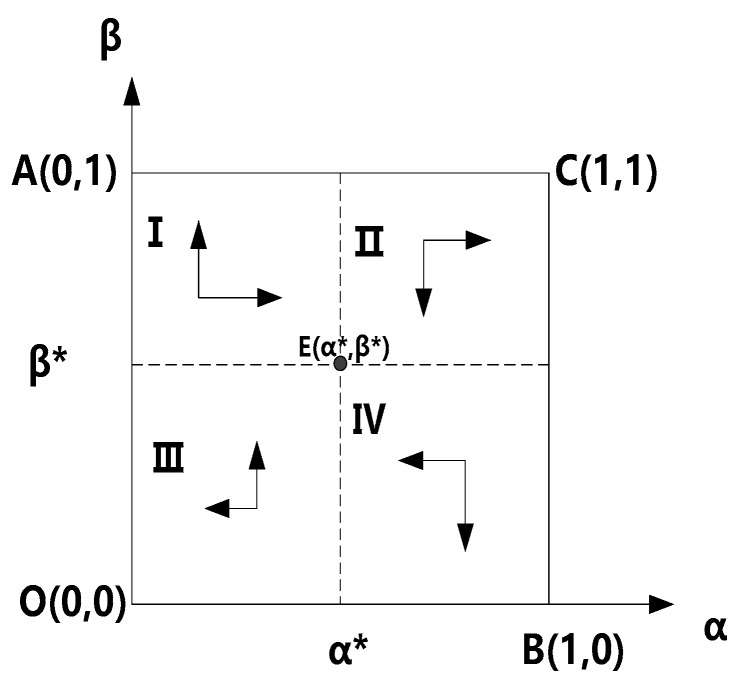
Phase portrait of the evolutionary game ($\beta^{*}=\frac{c_{c}-ap}{\left(1-a\right)p+bc_{s}},\alpha^{*}=1-\frac{c_{s}-p}{bc_{s}-ap}$).

Let Equation (3) equal 0, that is dα/dt=0, then we can obtain three zero points, such as $\alpha =0,\alpha =1,\beta^{*}=\left(c_{c}-ap\right)/\left(\left(1-a\right)p+bc_{s}\right)$. Furthermore, we will judge whether the above-mentioned points (including, α=0, α=1) is the equilibrium point. Specifically, if $\beta <\left(c_{c}-ap\right)/\left(\left(1-a\right)p+bc_{s}\right)$ we can conclude $dF\left(\alpha \right)/d\alpha {|}_{\alpha =0}<0,dF\left(\alpha \right)/d\alpha {|}_{\alpha =1}>0$, then α=0 is the equilibrium point, that is, schools tend to select the “no caution” strategy as their optimal strategy, namely, the evolutionary stability strategy (ESS). And if $\beta >\left(c_{c}-ap\right)/\left(\left(1-a\right)p+bc_{s}\right)$, then $\frac{dF\left(\alpha \right)}{d\alpha }{|}_{\alpha =0}>0$, $dF\left(\alpha \right)/d\alpha {|}_{\alpha =1}<0$, so we can conclude that α=1 is the equilibrium point, that is, the school tends to choose the “appropriate caution” strategy as their optimal strategy, namely, the evolutionary stability strategy (ESS). Similarly, let Equation (6) equal to 0, that is dβ/dt=0, then can also obtain three zero points, such as $\beta =0,\beta =1,\alpha^{*}=\left[\left(1-a\right)p-\left(1-b\right)c_{s}\right]/\left(bc_{s}-ap\right)$. Furthermore, we will judge whether those zero points (including, =0, β=1) is the equilibrium point. Specifically, if $\alpha <\left[\left(1-a\right)p-\left(1-b\right)c_{s}\right]/\left(bc_{s}-ap\right)$ then $dG\left(\beta \right)/d\beta {|}_{\alpha =0}>0$, $dG\left(\beta \right)/d\beta {|}_{\alpha =1}<0$, so we can conclude that β=1 is the equilibrium point, that is, the students and their guardians tend to select the “appropriate caution” strategy as their optimal strategy, namely, the evolutionary stability strategy (ESS). Additionally, if $\alpha >\left[\left(1-a\right)p-\left(1-b\right)c_{s}\right]/\left(bc_{s}-ap\right)$ then $dG\left(\beta \right)/d\beta {|}_{\alpha =0}<0$, $dG\left(\beta \right)/d\beta {|}_{\alpha =1}>0$, we can also conclude that β=0 is the equilibrium point, that is, the students and their guardians tend to select the “no caution” strategy as their optimal strategy, namely, the evolutionary stability strategy (ESS). The evolutionary stable region figure is presented in Figure 1.

As shown in Table 3 and Figure 1, when the prevention costs of the school, students, and their guardians are too high, $\left(\alpha^{*},\beta^{*}\right)$ will continuously decrease to zero with time. That is, when one of the game agents chooses the “no caution” strategy, both players will select the “no caution” strategy as the optimal strategy (see scenarios 1–4 in Table 3). In fact, Figure 1 has also demonstrated this result, namely, when the initial strategy of all game agents in the game is in region III, $E\left(\alpha^{*},\beta^{*}\right)$ will move to the lower-left corner and eventually converge to O(0,0). Additionally, when the cost of an accident loss is high, $E\left(\alpha^{*},\beta^{*}\right)$

$E\left(\alpha^{*},\beta^{*}\right)$ will increase with time until 1. That is, when one of the game agents selects the “appropriate caution” strategy, both players will choose the “appropriate caution” strategy as the optimal strategy (see scenarios 5–6 in Table 3). According to Figure 1, if the initial strategy of the two participants in the game is in region II, $E\left(\alpha^{*},\beta^{*}\right)$ will move to the upper-right corner and eventually converge to C(1,1). Furthermore, when $\pi_{2}<0,\pi_{4}>0$, that is, if students and their guardians adopt the “appropriate caution” strategy, the net income of the school of adopting the “appropriate caution” strategy is negative, but students’ income from the “appropriate caution” strategy would increase gradually. In this context, the “appropriate caution” strategy is not the school’s optimal strategy, while it is the student’s optimal strategy. Thus, the system would converge to the evolutionary stability state as {no caution, appropriate caution} (see scenarios 7–8 in Table 3) under this condition. According to Figure 1, when the initial strategy of the two parties in the game is in region I, $E\left(\alpha^{*},\beta^{*}\right)$ will move to the upper-left corner and finally converge to A(0,1). For the fourth scenario, that is, when the system satisfies $\pi_{1}>0,\pi_{3}<0$, when the school adopts the “appropriate caution” strategy, the net income of the students and their guardians of adopting the “appropriate caution” strategy is negative, and the school’ income from the “appropriate caution” strategy increases gradually. In this context, the “appropriate caution” strategy is not the student’s optimal strategy, while it is the school’s optimal strategy. Thus, this system converges to the evolutionary stability state as {appropriate caution, no caution} (see scenario 9 in Table 3) under this condition. According to Figure 1, when the initial strategy of the two parties in the game is in region IV, $E\left(\alpha^{*},\beta^{*}\right)$ will move to the lower-right corner and finally converge to B(1,0).

### 4.3. Numerical Simulation Analysis of Evolutionary Game System

**Figure 2:**
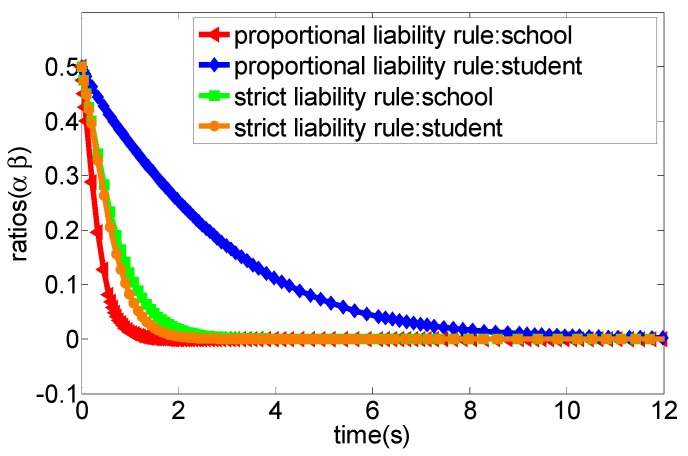
The evolution process of the system game strategy under the condition of high prevention cost from both parties (case 1).

**Figure 3:**
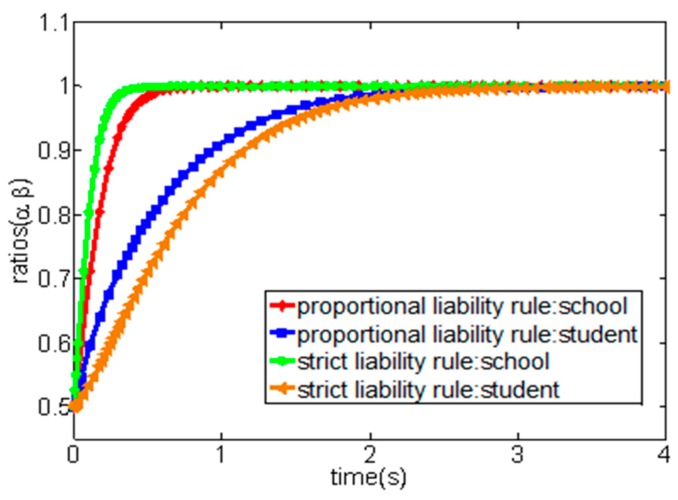
The evolution process of the system game strategy under the condition of high ratios of the prevention cost that school should undertake (case 2).

**Figure 4:**
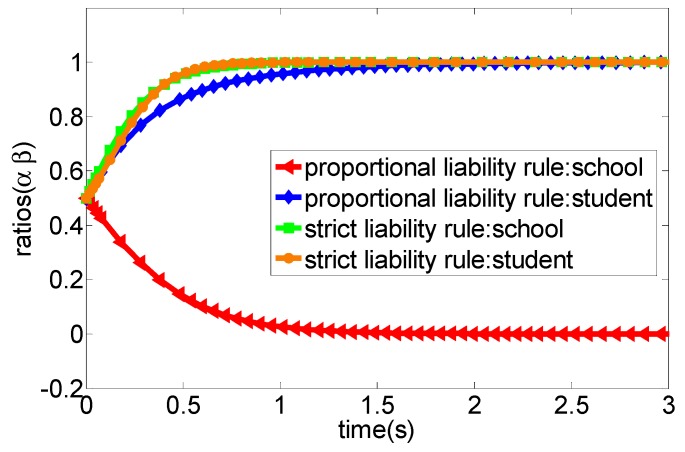
The evolution process of the system game strategy under the condition of high accident loss (case 3).

**Figure 5:**
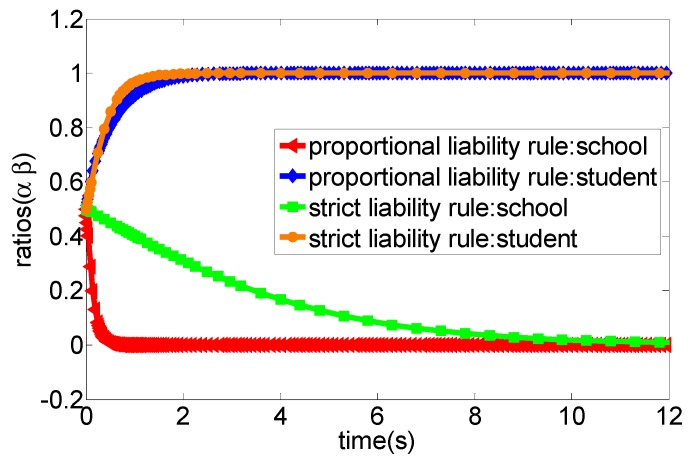
The evolution process of the system game strategy under the condition of low prevention cost from the student and high accident loss (case 4).

**Figure 6:**
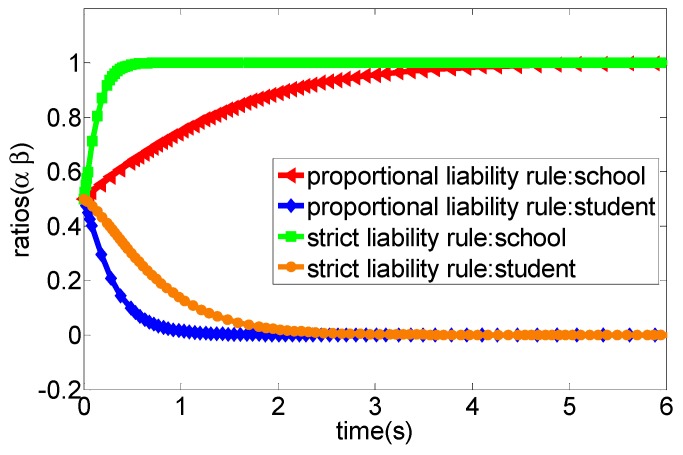
The evolution process of the system game strategy under the condition of low prevention cost from school and high accident loss (case 5).

After the Tort Liability Law of the People’s Republic of China [36] comes into force, the main principles for liability determination of school sports injury accidents are based on the principle of presumption of fault in article 38 and the principle of fault liability in article 39, of which article 38 emphasises that the school will undertake strict tort liability when the school cannot prove that the school is not at fault in sports injury accidents. Article 39 has three scenarios, firstly, the school will undertake all liability when the school is at fault, and students and their guardians are not at fault. Secondly, if the school is not at fault, and students and their guardians are negligent, students and their guardians would undertake all liability. Three, when the school is at fault, and students and their guardians are also at fault; they are liable according to their respective fault proportions [37]. Thus, to clearly describe the game process of sports injury accidents in schools, this section uses MATLAB R2012a (MathWorks, Inc., Natick, MA, USA) to simulate the strategy choices of both participants under different conditions. The specific evolutionary processes are shown in Figure 2, Figure 3, Figure 4, Figure 5 and Figure 6.

(1) According to the legal practice and the specific conditions of the game scenario, we set the related parameters of the system as $a\in \left[0,1],b\in [0,1\right],p=5,c_{c}=10,c_{s}=6$ and also set the initial evolutionary strategy ratio of the two participants in the game as (0.5, 0.5). The evolutionary result is presented in Figure 2. More specifically, when the cost of accident prevention in schools, students and their guardians is high, no matter what kind of liability principle is adopted to determine the liability, the two participants will gradually converge to the evolutionary stability strategy (no caution, no caution) as the costs expand.

(2) We set the relevant parameters as $a\in \left[0,1],b\in [0,1\right],p=12,c_{c}=8,c_{s}=10$, and set the initial evolutionary strategy probability of both parties of the game as (0.5, 0.5). As shown in Figure 3, when the proportion of the prevention cost of students and their guardians undertake by the school (b) is very high, no matter what principle the court adopts to determine the accident liability, all participants of the game would eventually converge to the evolutionary stability strategy as (appropriate caution, appropriate caution).

(3) We set the related parameters of the model as $a\in \left[0,1],b\in [0,1\right],p=12,c_{c}=8,c_{s}=4$. Figure 4 shows that the court adopts the strict liability rule for accident liability determination, the optimal strategy of both participants would eventually converge to the evolutionary stability state as (appropriate caution, appropriate caution) when the accident loss is relatively high. However, when the court adopts the proportional liability rule for accident liability determination, the optimal strategy of both participants would eventually converge to the evolutionary stability state as (no caution, appropriate caution).

(4) We set the model parameters as $a\in \left[0,1],b\in [0,1\right],p=12,c_{c}=16,c_{s}=4$. The evolutionary results are presented in Figure 5, that is, when the students’ net income from the “appropriate caution” strategy gradually increases, no matter what kind of liability principle the court adopts to determine the accident liability, the optimal strategy of both participants would eventually converge to the evolutionary stability state as (no caution, appropriate caution).

(5) Similarly, we set the model parameters as $a\in \left[0.5,1\right],\lambda =1/100,p=1000,c_{c}=400,c_{s}=1000$. According to Figure 6, when the income of the school of adopting the “appropriate caution” strategy gradually increases, no matter what kind of liability principle the court adopts to determine the liability, the optimal strategy of both participants would eventually converge to the evolutionary stability state as (appropriate caution, no caution).

Table 4 shows the evolution analysis results of the above cases.

## 5. Game Analysis of the Imputation of School Sports Injury Accidents

According to the above-mentioned model analysis, we can conclude that those parameters, including $c_{c},c_{s},a,b,p$, play an important role in affecting the strategic choice of both game agents. In this section, we will analyze the behavior patterns of the two parties in the school sports injury accidents under different liability principles.

### 5.1. Imputation Game Analysis under the Strict Liability Rule

According to the Tort Liability Law of the People’s Republic of China, the school should undertake all liability for school sports injury accidents under the guidance of the strict liability rule. More specifically, when students and their guardians adopt the “appropriate caution” strategy, the school can obtain the net income of $-c_{c}$ and $-p-c_{s}$ with “appropriate caution” and “no caution” strategies, respectively, of which $-c_{c}$ refers to the cost of school to prevent injury accidents, and $-p-c_{s}$ refers to the sum of the total loss of the accident and the prevention cost of the student. Additionally, when students and their guardians take “no caution” strategy, the net profit of school of adopting “appropriate caution” and “no caution” strategies are $-c_{c}-p$ and −p, respectively, of which −p refers to the total loss of the accident and $-c_{c}-p$ refers to the sum of the total loss of the accident and the prevention cost of the school. 

Obviously, the school would undertake all liability for the sports injury accident under the strict liability rule, while students and their guardians do not have to bear the liability of accidents regardless of the strategy adopted. In this context, students and their guardians, as rational persons, often tend to adopt the “no caution” strategy to avoid accident prevention costs, the school still undertakes the greater accident cost though it adopts the “appropriate caution” strategy in the progress of campus injury accidents and, thus, it would tend to choose the “no caution” strategy as an optimal strategy under this principle of imputation. This means that the school will have no incentive to invest enough funds in preventing sports injury accidents. The simulation analysis depicted in Figure 2 verified this situation. More specifically, when the prevention cost of the school is greater than the accident cost under the strict liability rule, then the strategy choice of both parties will eventually converge to the evolutionary stability state as (no caution, no caution). For example, in a primary school, a student broke their tibia and ribs caused by a football activity with a teacher during the break. The parents of the injured student strongly claimed that the school and the teacher should undertake all liability. As a consequence, the school finally compensated 57,000 yuan, and the teacher was dismissed [38]. Another example was that a school hires a retired teacher to teach basketball. A student suffered from a fracture in the left arm due to the slippery venue and insufficient warm-up before class. Similarly, the parents also urged that the school and teachers should undertake the main liability, and the court decided that the school compensate the students 30,000 yuan. Additionally, the teacher was also punished with dismissal [39]. In fact, the above-mentioned two accidents are not serious cases of sports injury accidents in schools. However, once the fatal accident occurs, the losses are very amazing. In summary, from the perspective of game theory, we can conclude that the strategy set (no caution, no caution) is the strict optimal strategy for both parties. However, in practice, due to the unpredictability of accident losses, schools are still likely to adopt appropriate caution strategies to prevent accidents, although this will generate some prevention costs. One important reason might be that the school’s strategy choice may change if the accident losses are high. For example, in Figure 4, we find that the school will tend to choose the appropriate caution strategy under the strict liability rule when the accident cost is high. Moreover, Figure 6 also shows that the increase of the net income from the appropriate caution strategy will cause the school to choose the appropriate caution strategy under the strict liability rule. In fact, students are also not completely rational economic humans, which means that students still may choose the appropriate caution strategy for moral consequences of possible deaths or permanent injuries under the strict liability rule.

### 5.2. Imputation Game Analysis under the Proportional Liability Rule

Under the guidance of the proportional liability rule, the court will decide the proportion of the liability of both parties according to whether the two parties ae at fault or not in the accident and the severity of the fault when a sports injury accident occurs in school. More specifically, from the above evolutionary game analysis, when students and their guardians adopt the “appropriate caution” strategy, the school can obtain the net income, such as $-c_{c}$ and $-p-bc_{s}$, respectively, when they choose “appropriate caution” and “no caution” strategies, of which $-c_{c}$ refers to the cost of school prevention injury accidents, and $-p-bc_{s}$ refers to the sum of the accident injury loss and the prevention cost (the prevention cost of students borne by the school according to the proportion of b). Additionally, when students and their guardians adopt the “no caution” strategy, the school can obtain the net income, such as $-c_{c}$ and −ap, respectively, when they select the “appropriate cautious” and “no caution” strategies, of which $-c_{c}$ refers to the cost of school prevention injury accidents, and −ap refers to the injury accidents cost borne by the school according to the proportion of a. Obviously, under the principle of the proportional liability rule, the court will determine the proportion of liability according to the principle of whether the parties are at fault or not and the severity of the fault. In this context, students may undertake the corresponding accident costs due to their faults in school sport injury accidents and, thus, they tend to select the “appropriate caution” strategy to prevent the occurrence of injury accidents. Additionally, the school also tends to choose the “appropriate caution” strategy as its best strategy based on its income considerations. In summary, both the school and students will jointly prevent sports injury accidents in this case and, thus, we can conclude that the proportional liability rule could encourage schools and students to adopt “appropriate caution” strategies to prevent sports injury accidents in schools, which is well reflected in Figure 3. More specifically, the strategy of both parties would gradually evolve into the strategy set as (appropriately cautious, appropriately cautious) overtime under the condition of the proportional liability rule. In fact, several cases can support this viewpoint. For example, Fang (2018) investigated 181 judicial precedents of sports injury accidents in primary and secondary schools in China in 2016, and indicated that schools undertake less than 50% of the liability in a sample of 56 cases where there was a clear infringer, while the school bore more than 50% of the liability in the 125 cases where there was no infringer was identified [40]. In summary, the strategy set of (appropriate caution, appropriate caution) is the proportional optimal strategy for both parties from the perspective of game theory but, in practice, when the income from the “appropriate caution” strategy is negative, the school may still tend to adopt the “no caution” strategy, which is well reflected in Figure 5.

### 5.3. Comparison of the Efficiency of the Two Principles

Through the above game analysis, we can conclude that both the strict liability rule and the proportional liability rule are effective principles of the imputation of school sports injury accidents under different conditions, which have good practical value for preventing accidents. However, in the specific judicial practice, there are differences between the strict liability rule and the proportional liability rule in imputation efficiency under a certain condition.

(1) Different standards regarding the proof

Under the strict liability rule, as long as students suffer sports injuries, the school must bear all liability regardless of the degree of caution of the school. In this principle, the court only needs to confirm whether the accident occurs within the scope of the management of the school. However, under the proportional liability rule, although it can promote both parties, such as the school and student, to select the appropriate caution strategy as their optimal strategy, the court must judge the proportion of liability that stakeholders need to undertake in the sport injury accident, which will, to some extent, increase its costs on evidence collection and verification in the process of accident fault identification. Additionally, the proportion judgment, in reality, mostly depends on the experience of judges and appraisers, which also increase the deviation for imputation. In summary, the imputation of school sports injury accidents under the proportional liability rule can help maximize social efficiency, but greatly increase the difficulty of proof for the court, which decreases the efficiency of imputation. On the contrary, although the strict liability rule fails to urge the school and students to adopt the appropriate caution strategy, it can increase the efficiency of imputation.

(2) Different incentive strengths

Under the strict liability rule, the “appropriate caution” strategy is not the optimal strategy for students and their guardians because they do not have to undertake the liability for sports injury accidents. That is, the school, as a rational actor, often tends to choose the “no caution” strategy as its optimal strategy. One important reason might be that the school must bear all liability of the accident no matter whether the students are cautious or not, which is well reflected in Figure 4 and Figure 6, More specifically, both high accident loss and low prevention cost from the school are the key factors of inducing the school take the “no caution” strategy. In this context, the school is more likely to invest less for preventing potential sports injury accidents. However, under the proportional liability rule, the liability of the accident is determined based on the degree of stakeholder’s fault. Thus, both parties have the strong motivation to choose the “appropriate caution” strategy as their optimal strategy because either party will undertake more liability once it chooses the “no caution” strategy, which is well reflected in Figure 5. In summary, both the school and students have no motivation to choose the “no caution” strategy, which means that the proportional liability rule can help standardize the behavior of both parties and, thus, can reduce the occurrence of sports injury accidents.

## 6. Discussion

In this article, we adopted an evolutionary game model to investigate the imputation progress in schools’ injury accidents and discuss the efficiency of two imputation principles in the imputation progress of school sports injury accidents. The results show that the cost of accident compensation and the prevention cost of both parties to the accident play a key role in the imputation progress of school sports injury accidents. Additionally, the proportional liability rule is more conducive in regulating the behavior of both parties and, thus, both schools and students tend to select (appropriate caution, appropriate caution) strategies to reduce sports injury accidents.

In the process of realizing accident liability both schools and students, as rational economic humans, can scientifically measure their gains and losses and, thus, can make a strategic choice. In this article, we find that both schools and students tend to take the “appropriate caution” strategy to prevent school sports injury accidents under the proportional liability rule. One important reason might be that the principle of “fault is a liability, no-fault is no liability” is the legal basis of the proportional liability rule, which means that the liability of school is a kind of limited liability, not unlimited liability [41]. In fact, If both parties assume the proportion of responsibility according to the actual liability situation in the sports injury accidents, it is conducive to achieving accountability remedies after an accident, and can help encourage both parties to take measures to lessen the likelihood of such accidents. This research result is consistent with the viewpoint of “taking the principle of fault liability as the basic principle” [20,22,23,24]. Additionally, many judicial cases also demonstrate it. According to a survey, there is 84.3% of school sports injury accidents are treated based on the fault liability principle. This means that the fault liability principle is the main applicable liability principle [42]. For example, in an 800-meter test of the sixth-grade physical education class of a primary school, several students merged into the same track and attempted to overtake. As a consequence, a collision occurred, which caused a fall injury to one student. However, the court judged that the school undertakes 70% of the liability, and students undertake 30% of the liability according to the proportional liability rule because the court thinks that both parties were at fault [43]. Another case of Kleinknecht, a student died of cardiac arrest in a practice session of a turf hockey team. The court judged that the school and the student undertake its corresponding liability, that of 50% of the liability since the school did not provide the required protective equipment, and students failed to confirm whether the protective equipment was in good condition before participating in the competition [44]. Additionally, this study also finds that participants’ strategy selections in the progress of imputation are mainly affected by the cost of accident prevention and the possible liability cost after the accident.

The strict liability rule means that the school should undertake all the liability when school sports injury accidents occur. Based on this principle, the court often thinks that the school, as a “strong” side, has a greater understanding of the characteristics of sports and the actual situation of sports venues and facilities and, thus, it has more powerful strength and professional ability to prevent students from sports injuries [27]. Therefore, this principle is an effective way to regulate the school’s behavior in sports injury accidents, as well as reduce the probability of occurrence of sports accidents. One important reason might be that the school often faces great economic compensation for sports injury accidents, the administrative accountability from government, and the great loss of reputation caused by public opinion. However, in practice, we can find that the school is likely to take measures to reduce or cancel students’ sports activities for avoiding the potential liability caused by sports accidents, rather than taking some preventative measure. In fact, this approach is not conducive to the healthy and comprehensive development of young students, and is not consistent with the current educational model advocated by the Chinese government.

## 7. Conclusions

Studies on school sports injury imputation have recently become a hot topic. The existing studies focus on investigating the liability determination in school sports injury accidents from a law perspective; few scholars explore this issue from the “law-economic” perspective. To fill this research gap, this paper aims to analyze the imputation progress in school sports injury accidents by using the method of evolutionary game theory. The main conclusions are as follows:

(1) If the accident prevention cost of school, students and their guardians are great, the game system would eventually converge to the evolutionarily stable state as (no caution, no caution), no matter what principle of attribution is adopted for liability determination. When the cost of school accident prevention decreases, the net benefit of the school’s “appropriate caution” strategy will gradually increase and, thus, the game system will eventually converge to the evolutionary stability state as (appropriate caution, no caution). Additionally, when the cost of sports accident prevention for students and their guardians reduce, which means that the net benefit of students and their guardians from the “appropriate caution” strategy will gradually increase, the game system would eventually converge to the evolutionary stability state as (no caution, appropriate caution). Furthermore, when the number of accident losses further increase, both participants in the game can obtain a gradual increase in positive income through “appropriate caution,” and, thus, the game system would eventually converge to the evolutionarily stable state as (appropriate caution, appropriate caution).

(2) Both the strict liability rule and the proportional liability rule can stimulate stakeholders to adopt “appropriate caution” strategies to prevent sports injury accidents, of which the strict liability rule focuses on the realization of the fair value of the law, while the proportional liability rule pays attention to the realization of the social efficiency of the law. In our article, we find that the orientation of fairness and efficiency should consider actual judicial practice. For example, when the court adopts the strict liability rule on a certain accident, it may further increase the liability of the side of the school, but it also causes the school to adopt negative accident prevention methods (such as reducing sports activities, limiting the difficulty of physical activity, etc.). These activities are not conducive to the development of school sports, but it can reduce the burden of proof from the weak, the cost of attribution of disputes, as well as the cost of causation determination. On the other hand, when the court adopts the proportional liability rule for attribution, the accident liability determination may face very large costs from collecting evidence, etc. In this context, it can further increase the social cost caused by accidents, but it is not conducive to maximizing social efficiency.

To sum up, evolutionary game theory provides a new reference idea for attribution liability for school sports injury accidents, as well as provides a new analytical framework for analyzing the behavior strategy choice of school sports accident-related subjects. In our article, we find that the liability determination of school sports injury accidents needs to synthesize many factors due to the particularity of school physical education. Additionally, we also find that the liability determination of school sports injury accidents should take the principle of fault liability as the main principle, and the fair liability as the auxiliary principle in China’s judicial practice. Yet, there are some limitations in this study. Firstly, this research hypothesizes all participants are rational economic humans and, thus, they can accurately measure the gains and losses of interests and select their optimal and rational strategy in the game. In fact, all participants’ legal attribution behavior is limitedly rational in the process of imputation, which means that each player is not fully rational, their strategy selections are affected by irrational factors, such as social environment, personal values, and so on. In this context, game theory fails to consider those irrational factors on strategy selections of game agents in the progress of imputation. Thus, in the future research about sports injury accident imputation, it is necessary to conduct a more detailed analysis of the individual cases, appropriately absorb more indicators, improve the model and optimize the analysis results. For example, moral consideration is an important factor since no one can ignore the moral consequences of death or permanent injuries, and select its strategy in the progress of imputation based on rational economic strategy. Second, a sports injury accident often involves more than two other subjects of liability, such as third-party infringers, including students, contractors of school sports facilities, and insurance companies. This paper did not discuss the more complex multi-party game.

## Figures and Tables

**Table 1:** The game matrix between the two participants.

Game Players		School	
		Appropriate Caution (α)	No Caution (1 − α)
Students and Their Guardians	Appropriate caution (β)	($-c_{s},-c_{c}$)	($-\left(1-b\right)c_{s},-p-bc_{s}$)
	No caution (1−β)	($-p,-c_{c}$)	(−(1−a)p,−ap)

**Table 2:** The determinant and trace of equilibrium points.

Equilibrium Point	Determinant	Trace
O(0,0)	$\left(ap-c_{c}\right)\left(\left(1-a\right)p-\left(1-b\right)c_{s}\right)$	$p-c_{c}-\left(1-b\right)c_{s}$
A(0,1)	$-\left(p+bc_{s}-c_{c}\right)\left(\left(1-a\right)p-\left(1-b\right)c_{s}\right)$	$ap+c_{s}-c_{c}$
C(1,0)	$-\left(ap-c_{c}\right)\left(p-c_{s}\right)$	$\left(1-a\right)p+c_{c}-c_{s}$
B(1,1)	$\left(p+bc_{s}-c_{c}\right)\left(p-c_{s}\right)$	$-2p+\left(1-b\right)c_{s}+c_{c}$

**Table 3:** Local stability analysis of equilibrium points (EP).

**EP**	**Scenario 1:** $\pi_{1}<0,\pi_{2}<0,\pi_{3}<0,\pi_{4}<0$					**Scenario 2:** $\pi_{1}<0,\pi_{2}>0,\pi_{3}<0,\pi_{4}<0$					
	**Determinant**		**Trace**		**Result**	**Determinant**	**Trace**		**Result**		
O (0,0)	+		-		ESS	+	-		ESS		
A (0,1)	-		~		Saddle	+	+		unstable		
C (1,0)	-		~		Saddle	-	~		Saddle		
B (1,1)	+		+		unstable	-	~		Saddle		
**EP**	**Scenario 3:** $\pi_{1}<0,\pi_{2}>0,\pi_{3}>0,\pi_{4}<0$					**Scenario 4:** $\pi_{1}<0,\pi_{2}<0,\pi_{3}>0,\pi_{4}<0$					
	**Determinant**		**Trace**		**Result**	**Determinant**	**Trace**			**Result**	
O (0,0)	+		-		ESS	+	-			ESS	
A (0,1)	+		+		unstable	-	~			Saddle	
C (1,0)	+		+		unstable	+	+			unstable	
B (1,1)	-		-		unstable	-	~			Saddle	
**EP**	**Scenario 5:** $\pi_{1}<0,\pi_{2}>0,\pi_{3}>0,\pi_{4}>0$					**Scenario 6:** $\pi_{1}>0,\pi_{2}>0,\pi_{3}>0,\pi_{4}<0$					
	**Determinant**		**Trace**		**Result**	**Determinant**	**Trace**		**Result**		
O (0,0)	-		~		Saddle	-	~		Saddle		
A (0,1)	-		~		Saddle	+	+		unstable		
C (1,0)	+		+		unstable	-	~		Saddle		
B (1,1)	+		-		ESS	+	-		ESS		
**EP**	**Scenario 7:** $\pi_{1}<0,\pi_{2}<0,\pi_{3}>0,\pi_{4}>0$					**Scenario 8:** $\pi_{1}<0,\pi_{2}<0,\pi_{3}<0,\pi_{4}>0$					
	**Determinant**	**Trace**		**Result**		**Determinant**		**Trace**			**Result**
O (0,0)	-	~		Saddle		-		~			Saddle
A (0,1)	+	-		ESS		+		-			ESS
C (1,0)	+	+		unstable		-		~			Saddle
B (1,1)	-	~		Saddle		+		+			unstable
**EP**	**Scenario 9:** $\pi_{1}>0,\pi_{3}<0,\pi_{4}>0$					****Scenario 10:**** $\pi_{1}\pi_{2}<0,\pi_{3}\pi_{4}<0,\pi_{1}\pi_{3}>0$					
	**Determinant**		**Trace**		**Result**	**Determinant**	**Trace**		**Result**		
O (0,0)	+		+		unstable	-	~		Saddle		
A (0,1)	-		~		Saddle	-	~		Saddle		
C (1,0)	+		-		ESS	-	~		Saddle		
B (1,1)	-		~		Saddle	-	~		Saddle		

**Table 4:** Results of the evolution of the system game strategy. (☆ is for the strict liability rule. ★ is for the proportional liability rule.)

	Strategy	(School: No Caution, Student/Guardians: No Caution)	(School: Appropriate Caution, Student/Guardians: Appropriate Caution)	(School: Appropriate Caution, Student/Guardians: No Caution)	(School: No Caution, Student/Guardians: Appropriate caution)
Case					
Case 1 ($c_{c}$ and $c_{s}$ are high)		☆ ★			
Case 2 (b is high)			☆ ★		
Case 3 (p is high)			☆		★
Case 4 ($-p-c_{s}$ increase)					☆ ★
Case 5 ($-p-c_{c}$ increase)				☆ ★	
